# Effect of computer aided detection device on the adenoma detection rate and serrated detection rate among trainee fellows

**DOI:** 10.1002/jgh3.70018

**Published:** 2024-09-09

**Authors:** Anas Khouri, Chance Dickson, Alvin Green, Abrahim Hanjar, William Sonnier

**Affiliations:** ^1^ Department of Internal Medicine and Division of Gastroenterology University of South Alabama Frederick P. Whiddon College of Medicine Mobile Alabama USA

**Keywords:** adenoma detection rate (ADR), artificial intelligence, computer aided detection device (CADe), serrated polyp detection rate (SDR)

## Abstract

**Background and Aims:**

The utilization of artificial intelligence (AI) with computer‐aided detection (CADe) has the potential to increase the adenoma detection rate (ADR) by up to 30% in expert settings and specialized centers. The impact of CADe on serrated polyp detection rates (SDR) and academic trainees ADR & SDR remains underexplored. We aim to investigate the effect of CADe on ADR and SDR at an academic center with various levels of providers' experience.

**Methods:**

A single‐center retrospective analysis was conducted on asymptomatic patients between the ages of 45 and 75 who underwent screening colonoscopy. Colonoscopy reports were reviewed for 3 months prior to the introduction of GI Genius™ (Medtronic, USA) and 3 months after its implementation. The primary outcome was ADR and SDR with and without CADe.

**Results:**

Totally 658 colonoscopies were eligible for analysis. CADe resulted in statistically significant improvement in SDR from 8.92% to 14.1% (*P* = 0.037). The (ADR + SDR) with CADe and without CADe was 58% and 55.1%, respectively (*P* = 0.46). Average colonoscopy (CSC) withdrawal time was 17.33 min (SD 10) with the device compared with 17.35 min (SD 9) without the device (*P* = 0.98).

**Conclusion:**

In this study, GI Genius™ was associated with a statistically significant increase in SDR alone, but not in ADR or (ADR + SDR), likely secondary to the more elusive nature of serrated polyps compared to adenomatous polyps. The use of CADe did not affect withdrawal time.

## Graphical abstract

The implementation of CADe in our study led to a significant increase in SDR without affecting the withdrawal time. There was a trend towards an increase in (ADR + SDR) but it did not reach statistical significance. Further and larger studies are required to determine the contexts where these devices can be helpful and to determine the patient's population that would benefit most from the implication of this technology.

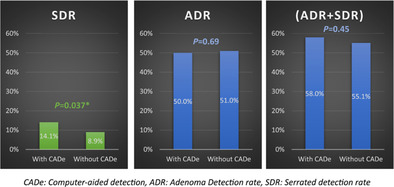

## Introduction

Colorectal cancer (CRC) is associated with significant morbidity and mortality, and it is the second most common cause of cancer‐related deaths among both men and women worldwide.^1^ Evidence has validated that routine screening via colonoscopy has decreased CRC incidence and mortality by detecting and removing precancerous polyps.^2^ Studies have demonstrated that lower‐quality colonoscopies have resulted in increased CRC rates due to the lower detection of precancerous polyps.^3^, ^4^ Therefore, Adenoma detection rate (ADR) and Serrated polyp detection rate (SDR) are considered important metrics of quality and care that represent the percentage of precancerous polyps' detection during routine screening colonoscopies. The US task force suggests an ADR benchmark of 25%.^5^

Adenoma miss rate (AMR) can lead to an increased rate of CRC. In a meta‐analysis conducted by Zhao et al., one‐fourth of colorectal neoplasms are missed during routine screening.^6^

Recent studies have shown that utilization of artificial intelligence (AI) with computer‐aided detection (CADe) may increase ADR, potentially decreasing CRC incidence and mortality.^7^, ^8^ One notable study by Repici et al. showed that the implementation of artificial intelligence (AI) with CADe can increase the ADR by up to 30% in expert settings and specialized centers.^9^ With every 1% increase in ADR, the risk of developing colon cancer over the next year decreases by 3%.^3^

Serrated polyps account for up to 30% of CRCs and are more difficult to detect because they are typically flat, translucent in color, and are mostly located in the proximal colon where inadequate bowel preparation can result in decreased visibility.^1^, ^10^

Withdrawal time (WT) is another important aspect of quality care during screening colonoscopies. An average withdrawal time of more than 6 to 9 min is considered a quality indicator for achieving higher ADR and lower interval CRC rates by gastroenterology societies including the American Gastroenterological association (AGA), American College of Gastroenterology (ACG), and the American Society for Gastrointestinal Endoscopy (ASGE).^11^, ^12^, ^13^ A study conducted by Butterly et al., showed that increased WT of 9 min resulted in nearly a 30% increase in SDR,^14^ indicating that longer WT could potentially increase SDR.

AI systems used to improve ADR have also led to inadvertent increases in WT.^15^ However, this remains controversial as other studies could not attribute the use of AI systems to prolonged colonoscopy withdrawal times.^16^

Analysis of AI with CADe on improving quality metrics in the setting of trainees with less colonoscopy experience remains limited. A retrospective study conducted by Huang et al. revealed that ADR is positively correlated with senior level endoscopists and single examiner colonoscopies,^17^ but this study did not include AI with CADe. Most studies that have analyzed the utilization of AI with CADe and its impact on quality measures are conducted in expert settings and specialized centers and do not analyze AI with CADe in settings that include trainees. In addition, there are limited data on the ability of AI systems to increase SDR as most studies have focused exclusively on increased ADR as the primary end point.

In our study, we aimed to investigate the reproducibility of prior results, in addition to the effect of AI on SDR, in an academic center that included trainees with less colonoscopy experience and determine if the benefits of CADe vary based on the level of experience.

**Figure 1:**
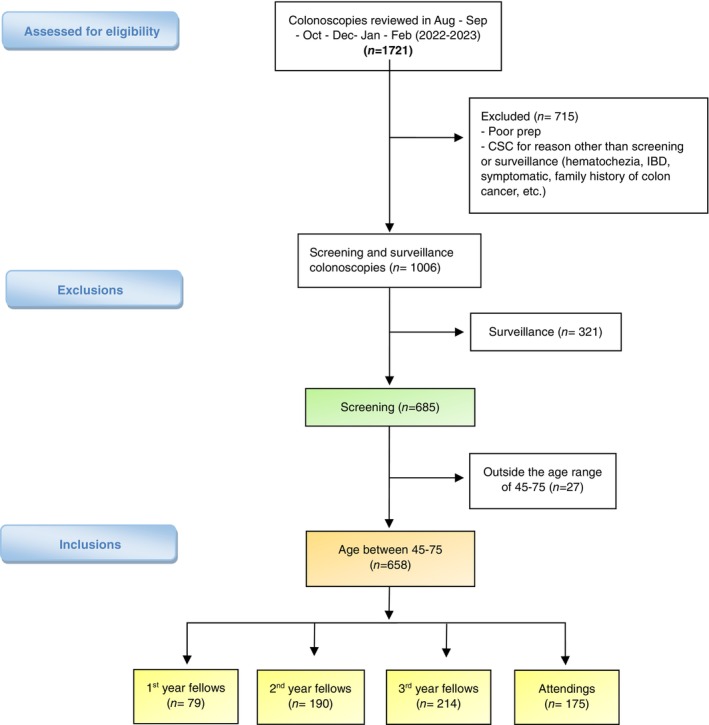
Flow chart of patients included and excluded in the retrospective study.

## Methods

This is a single‐center retrospective observational study conducted at the University of South Alabama Health University Hospital. We retrieved the medical records of all patients who underwent colonoscopy 3 months prior to implementation of GI Genius™ (Medtronic, USA) (Aug, Sep, Oct) and 3 months of CSC data after implementation (Dec, Jan, Feb). We collected data regarding whether the colonoscopy was performed with or without the device, provider experience (first year fellow, second year fellow, third year fellow, attending), patient demographics, age at time of colonoscopy, colonoscopy indication, FIT test or Cologuard status, bowel preparation quality, withdrawal time (including polypectomies), number and type of polyps removed, and pathology report. Inclusion criteria were asymptomatic screening CSC performed on patients between the ages of 45 and 75. Exclusion criteria were patients less than 45 or more than 75 years old, patients with higher than average risk (family history of colon cancer), CSC performed for any diagnostic or therapeutic purposes, and CSC with inadequate bowel preparation (Fig. 1). ADR is calculated by dividing the number of colonoscopies in which one or more adenomas are detected by the total number of colonoscopies performed by the endoscopist. Similarly, SDR is calculated by dividing the number of screening colonoscopies in which one or more serrated polyps are detected by the total number of colonoscopies. SDR has been shown to highly correlates with ADR.^18^

ADR/SDR was recorded if an adenomatous polyp or sessile serrated polyp (SSP) was confirmed on the pathology report. The detection rate was stratified by the training level. The primary outcome was ADR and/or SDR with and without CADe. Secondary outcome was withdrawal time with and without the device. Ten investigators participated in the data collection. IRB approval was exempted for this study as this was not a prospective study and did not meet the definition of “clinical trial” by the ICMJE. The analysis involved de‐identified data.

Statistical analysis: categorical variables were reported in frequencies and percentages and compared using the Pearson Chi‐Square test (*χ* ^2^). Continuous variables were reported as means and standard deviations and compared using t‐test and Wilcoxon rank sum tests or analysis of variance (ANOVA). The non‐parametric Kruskal–Wallis test was used to assess the medians of withdrawal time across provider experience, as that data did not meet normality assumptions for ANOVA. Statistical significant values across more than two groups were assessed using residual values for chi‐square test, and Turkey HSD test for ANOVA/Kruskal–Wallis to estimate where the statistical significance is originating from. The statistical analysis was performed using the JMP statistical package. A *P*‐value <0.05 was considered statistically significant.

## Results

Totally 1721 CSC were assessed for eligibility. After application of inclusion and exclusion criteria, 658 CSC were eligible for analysis (Fig. 1). Among the 658 patients included, there were 295 (45%) males and 363 (55%) females. The average age of our study population was 57.4 years (SD ± 8). A similar number of patients were in the “with CADe” and “without CADe” groups with 333 (50.6%) CSC with and 325 (49.4%) CSC without the GI Genius™ device, respectively. First‐year fellows' group had less CSC compared to the more advanced fellows and attendings groups with 79 (12%) CSC. 190 (29%), 214 (32.5%), and 175 (26.5%) CSC were performed by the second year fellow, third year fellow, and attending groups, respectively. Overall, the ADR + SDR rate was 56.5% (Table 1).

**Table 1:** Characteristics of the study population

Group distribution (*N*, %)	
With the genius device (CADe)	333, 50.6%
Without the genius device (CADe)	325, 49.4%
Gender (*N*, %)	
Male	295, 45%
Female	363, 55%
Age (Mean, SD)	57.4 (8)
Provider's experience (*N*, %)	
First year fellows	79, 12%
Second year fellows	190, 29%
Third year fellows	214, 32.5%
Attendings	175, 26.5%
Withdrawal time in minutes (Mean, SD)	17.3 (9.6)
Detection rate (*N*, %)	
ADR	333, 50.6%
SDR	76, 11.5%
Total ADR + SDR	372, 56.5%

ADR, Adenoma Detection rate; CADe, Computer‐aided detection; SDR, Serrated detection rate.

There was no statistically significant difference in gender (*P* = 0.32), age (*P* = 0.22), or FIT test status (*P* = 0.08) across both “with CADe” and “without CADe” groups. The provider experience across both groups differed with second year fellows having more colonoscopies without CADe compared to first year, third year, and attendings who had more colonoscopies with CADe (*P* = 0.002) (Table 2).

**Table 2:** Demographics and results with their associated statistical significance across both groups

	Without CADe	With CADe	*P*‐value
Gender (*N*, %)			0.32
Males	152 (47%)	143 (43%)	
Females	173 (53%)	190 (57%)	
Age (Mean, SD)	57.8 (8)	57 (8)	0.22
Positive FIT test prior to colonoscopy (*N*, %)	24 (7.4%)	14 (4.2%)	0.08
Provider experience			0.002*
First year fellows	33 (10%)	46 (14%)	
Second year fellows	115 (35%)	75 (22%)	
Third year fellows	102 (32%)	112 (34%)	
Attendings	75 (23%)	100 (30%)	
Withdrawal time (Mean, SD)	17.3 (9)	17.3 (10)	0.98
Detection rate			
ADR	51.4%	49.9%	0.7
SDR	8.92%	14.1%	0.037*
Total ADR + SDR	55.1%	58%	0.46

* Statistically significant (*P* < 0.05).

ADR, Adenoma detection rate; CADe, Computer‐aided detection; FIT, Fecal immunochemical test; SDR, Serrated detection rate.

### Primary outcomes

**Figure 2:**
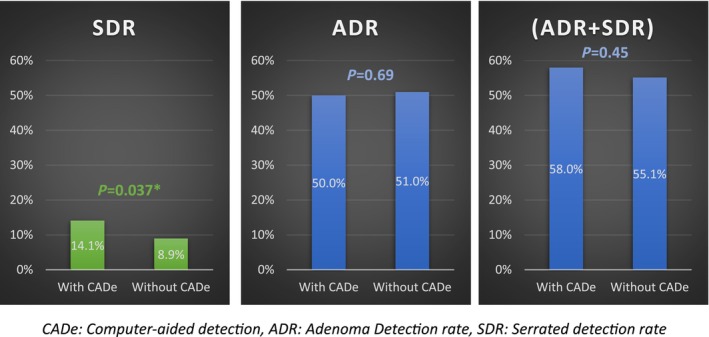
ADR + SDR with and without CADe. CADe, Computer‐aided detection, ADR, Adenoma Detection rate, SDR, Serrated detection rate.

There was no significant difference in (ADR + SDR) with and without CADe (58% vs. 55.1%, *P* = 0.45). (Fig. 2). When the results were stratified by provider experience and training level, first year fellows had the largest increase in their ADR + SDR when using CADe. However, similar to the overall analysis, this increase was not statistically significant (54.3% vs. 45.4%, *P* = 0.44) (Table 3). On the other hand, CADe resulted in statistically significant increase in the SDR which improved from 8.92% to 14.1% (*P* = 0.037). This increase was universal across all levels of training when stratified by the provider's experience (Table 4). This increase was not found in the ADR alone or the ADR + SDR as mentioned above.

**Table 3:** (ADR + SDR) stratified by provider experience

	ADR + SDR without CADe	ADR + SDR with CADe	*P*‐value
First year fellows (*n* = 79)	45.4%	54.3%	*P* = 0.43
Second year fellows (*n* = 190)	48.7%	50.7%	*P* = 0.79
Third year fellows (*n* = 214)	64.7%	64.3%	*P* = 0.95
Attendings (*n* = 175)	56%	58%	*P* = 0.79
Overall (*n* = 658)	55.1%	58%	*P* = 0.46

ADR, Adenoma detection rate; CADe, Computer‐aided detection; SDR, Serrated detection rate.

**Table 4:** SDR stratified by provider experience

	SDR without CADe	SDR with CADe	*P*‐value
First year fellows (*n* = 79)	9.1%	13%	0.58
Second year fellows (*n* = 190)	4.3%	9.3%	0.17
Third year fellows (*n* = 214)	12.8%	17.9%	0.3
Attendings (*n* = 175)	10.7%	14%	0.5
Overall (*n* = 658)	8.9%	14.1%	0.037*

ADR, Adenoma Detection rate; CADe, Computer‐aided detection; SDR, Serrated detection rate.

### Secondary outcome

There was no significant difference in the average withdrawal time with and without CADe (17.33 vs. 17.35 min, *P*‐value 0.98). There were notable differences in the withdrawal time across the provider experience groups which inversely correlated with the training level. Median withdrawal time was 19.5 min for first year fellows, compared to 17 min for second year fellows, 14 min for third year fellows, and 12 min for the attending. This was statistically significant (*P*‐value <0.0001) (Table 5).

**Table 5:** Withdrawal time stratified by provider experience with and without CADe

	Without CADe	With CADe	P‐value
First year fellows (*n* = 79)			
Mean (SD)	20 (8)	23 (10)	0.16
Median (IQR)	20.5 (14–25)	19 (17–27.5)	
Second year fellows (*n* = 190)			
Mean (SD)	21 (10)	19 (9)	0.15
Median (IQR)	18.5 (13–24.25)	16 (12–24.25)	
Third year fellows (*n* = 214)			
Mean (SD)	14.6 (8)	17.7 (12)	0.22
Median (IQR)	14 (9–18)	14 (9.75–21.25)	
Attendings (*n* = 175)			
Mean (SD)	14 (5)	13 (5)	0.25
Median (IQR)	13 (10–17)	12 (9–15)	
Overall (*n* = 658)			
Mean (SD)	17.35 (9)	17.33 (10)	0.98
Median (IQR)	15 (11–21)	15 (10.75–21)	
Overall median time across training level	First year fellows: 19.5 (15.25–26)		0.0001*
	Second year fellows: 17 (13–24)		
	Third year fellows: 14 (9–20)		
	Attendings: 12 (10–15.75)		

* Statistically significant (*P* < 0.05).

ADR, Adenoma Detection rate; CADe, Computer‐aided detection; SDR, Serrated detection rate.

## Discussion

Our study provides additional data regarding the promising integration of artificial intelligence with endoscopy. It included a substantial number of colonoscopies (658) helping provide validity to our data. In addition to the large volume of colonoscopies analyzed, focusing on screening colonoscopies makes our research particularly relevant in understanding the benefits of AI in the screening population.

The available data on the role of CADe in enhancing SDR are limited, and consensus regarding its benefits remains inconclusive. Some studies showed a statistically significant improvement in SDR while others did not.^19^, ^20^, ^21^ The implementation of GI Genius™ at our institution led to a substantial and a statistically significant increase in SDR which was seen across all providers' experience.

While the trend toward improvement in ADR + SDR was not statistically significant, it indicates a positive direction that might become significant with a larger sample size or longer study duration. One potential theory for the more apparent increase in SDR could be the inherent difficulty in finding serrated polyps during colonoscopy which has a proven high variable detection rate dependent on the endoscopist performing the procedure.^22^ Utilizing a polyp identification tool like GI Genius™ might help alleviate some of the endoscopist dependent variability seen especially while endoscopists are still in fellowship.^23^ It is important to note that serrated polyp has a high rate of progression to carcinoma.^24^ Given the deleterious results of missing a serrated polyp, any increase in SDR might be worth the effort.

Our study hinted that less experienced fellows might benefit more from the assistance of CADe technology as they develop their skills. However, we could not draw any absolute conclusion regarding that due to the lower number of colonoscopies performed by first year fellows compared to their peers, and other confounding factors like their growing experience as they advance in their training from August to February. This improvement in ADR with the advancing fellow experience has been well documented in the literature.^25^ Further studies need to be done at training centers to delineate the precise impact of CADe on different levels of training, and to optimize the integration of CADe in training programs.

Our study provided reassurance that using CADe did not increase the duration of a colonoscopy. Therefore, its use should not be hindered by concerns for increased procedural time and its associated risk to the patient and increasing costs.

The overall strengths of our study include looking at implementing CADe at a teaching institution across all levels of training which allows us to understand the different effects of implementation at different levels of training. This highlights the known variability between individual endoscopists. The less experienced the endoscopist, the greater their potential benefit from CADe as they are more likely to miss polyps until they have performed a certain number of procedures under direct supervision.^25^

The limitations of our study include the retrospective nature of the study design. A randomized control trial would have reduced bias through randomization. However, given the nature of the GI Genius interface, blinding the endoscopist would be challenging. In addition, given the variability of trainee schedules, there is some concern that the small sample size of colonoscopies performed by the first‐year fellows might have influenced the possible improvement seen with ADR in first year fellows while using CADe. In addition, our study was at a single center, therefore possibly limiting its extrapolation to a larger population. Finally, our study did not consider polyp size or location, which very well might be factors associated with CADe providing more benefit in those specific situations. Future studies should include these variables to provide a more comprehensive assessment of CADe performance.

## Conclusion

In our training institution, the implementation of CADe led to a significant increase in SDR, potentially due to sessile nature of the polyps, rendering them more elusive to detection. The use of CADe did not affect withdrawal time. Although there was an increase in (ADR + SDR) for both the fellows and attendings, this increase did not reach statistical significance.

The integration of artificial intelligence for computer‐assisted detection of adenomas does not necessarily enhance the detection rates in all settings. Further and larger studies are required to determine the contexts where these devices can be helpful and to determine the patient's population that would benefit most from the implication of this technology.
